# Challenges for Therapeutic Applications of Opsin-Based Optogenetic Tools in Humans

**DOI:** 10.3389/fncir.2020.00041

**Published:** 2020-07-15

**Authors:** Yi Shen, Robert E. Campbell, Daniel C. Côté, Marie-Eve Paquet

**Affiliations:** ^1^Department of Chemistry, University of Alberta, Edmonton, AB, Canada; ^2^Department of Chemistry, Graduate School of Science, The University of Tokyo, Tokyo, Japan; ^3^Centre de Recherche CERVO, Université Laval, Quebec City, QC, Canada; ^4^Département de Physique et Génie Physique, Université Laval, Quebec City, QC, Canada; ^5^Département de Biochimie, Microbiologie et Bioinformatique, Université Laval, Quebec City, QC, Canada

**Keywords:** optogenetics, viral vectors, therapeutic applications, technical challenges, opsins

## Abstract

As the technological hurdles are overcome and optogenetic techniques advance to have more control over neurons, therapies based on these approaches will begin to emerge in the clinic. Here, we consider the technical challenges surrounding the transition of this breakthrough technology from an investigative tool to a true therapeutic avenue. The emerging strategies and remaining tasks surrounding genetically encoded molecules which respond to light as well as the vehicles required to deliver them are discussed.The use of optogenetics in humans would represent a completely new paradigm in medicine and would be associated with unprecedented technical considerations. To be applied for stimulation of neurons in humans, an ideal optogenetic tool would need to be non-immunogenic, highly sensitive, and activatable with red light or near-infrared light (to maximize light penetration while minimizing photodamage). To enable sophisticated levels of neuronal control, the combined use of optogenetic actuators and indicators could enable closed-loop all-optical neuromodulation. Such systems would introduce additional challenges related to spectral orthogonality between actuator and indicator, the need for decision making computational algorithms and requirements for large gene cassettes. As in any gene therapy, the therapeutic efficiency of optogenetics will rely on vector delivery and expression in the appropriate cell type. Although viral vectors such as those based on AAVs are showing great potential in human trials, barriers to their general use remain, including immune responses, delivery/transport, and liver clearance. Limitations associated with the gene cassette size which can be packaged in currently approved vectors also need to be addressed.

## Introduction

Therapeutic applications of optogenetic techniques, which take advantage of the exquisite levels of cellular control that are enabled using the combination of light and genetic targeted constructs, are increasingly plausible. In particular, the ambitious goal to cure diseases of the nervous system would take a substantial step forward if researchers and clinicians were empowered to safely introduce and control optogenetic tools in humans. While there are several different classes of optogenetic tools (e.g., LOV domains, phytochromes, photocleavable proteins; Rost et al., 2017; Zhang et al., 2017), genetically encoded opsins (light-activated ion channels or pumps; Zhang et al., 2006; Deisseroth, 2015), are of the most relevance for therapeutic control of the nervous system. Optogenetic opsins are proteins that were borrowed from various microbial species and re-engineered or otherwise adapted for mammalian expression. Illumination with the appropriate wavelength of light allows ions to flow (or be actively pumped) across the membrane, leading to reversible activation or inhibition of a neural cell. Although not the focus of this review article, it is important to mention the existence of chemogenetic tools, which are genetically-encoded receptors that can activate or inhibit neurons upon small molecule agonists binding (Magnus et al., 2019). This approach offers significant therapeutic potential with the advantage of targeting that comes with gene therapy and the convenience of pharmacology. While optogenetics may have to overcome challenges of light delivery, the added spacial control and time resolution are critical components of this transformative technology.

A key stepping stone towards bringing optogenetic tools to the clinic will be intensive testing and validation in non-human primates (NHP). To date, there have been relatively few reports of using optogenetics in NHP (Galvan et al., 2017), likely due to both cost constraints and ethical considerations. Given the limited examples reported to date, it is apparent that open data sharing within the academic community will be an important aspect of moving the field forward. To help facilitate data sharing, researchers at the University of Pennsylvania are leading an initiative to put together a database of positive and negative results for various studies involving optogenetics in different species of NHP (Tremblay, S., NHP Optogenetics Open Database, retrieved from osf.io/mknfu August 7, 2019). This effort has already helped to highlight the very large number of variables and the many technical challenges that come with the use of this technology in larger species. Addressing these issues will advance primate neuroscience research and further translation to human medicine.

Here, we consider the technical challenges yet to be overcome to translate optogenetics from a tool for the investigation of model organisms to a therapeutic for the treatment of human diseases. The use of optogenetic tools for human gene therapies would represent a completely new paradigm in medicine and is associated with a combination of challenges, some of which are novel and some of which have precedent in the development of other clinical treatments. For example, precedent for aspects of optogenetic therapies can be found in gene therapy, and chronic brain implants used for Deep Brain Stimulation (DBS). An example of an entirely novel aspect would be light delivery deep into the tissue. We discuss a select number of these challenges that researchers from the fields of protein engineering, optics, genetics, virology, process optimization, and even economics will need to address to bring the therapeutic potential of optogenetics closer to patients.

At a minimum, to be applied for stimulation of neurons in human patients, an ideal optogenetic therapy would require: (1) a safe and efficient gene delivery vehicle; (2) Targeting of the gene delivery vehicle to the tissue of interest; (3) a delivery vehicle, transgene, and therapeutic protein gene-product, that is non-immunogenic and non-mutagenic; and (4) an optogenetic protein that is highly sensitive to light in the red to near-infrared wavelength range (to keep light doses low, maximize light penetration, and minimize photodamage). Additionally, the delivery of light itself also becomes a major issue when dealing with humans and primates compared to more commonly used animal models such as rodents. Overall, the large size of the primate brain, and the human brain, in particular, means that strategies optimized in mice models will need to be entirely rethought and redesigned.

## Near-Future Prospects for Clinical Applications of Optogenetics

Clinical applications for optogenetics are diverse but the field of vision restoration has shown particular promise with two clinical trials already ongoing (NCT02556736; NCT03326336). Many of the hurdles discussed in this review article are diminished in the case of treating retinal degeneration, which is the cause of most cases of blindness. Indeed, the affected cells are accessible to both light and transgene delivery, which has already contributed to the success of optogenetics to restore light sensitivity in various species (Baker and Flannery, 2018). Another promising application area is the treatment of severe epilepsy (Walker and Kullmann, 2020). In this case, traditional gene therapy, which is based on the replacement of a defective gene with a functional one, is associated with complications due to issues of dosage. Indeed, gene expression levels are difficult to control but the use of light to activate a genetically encoded channel provides a “dosage dial” that can be turned up or down as need be. There is also hope that optogenetics may replace the traditional electrode-based cochlear implants used to treat certain forms of hearing loss. Although electrical stimulation has been used extensively and successfully in the cochlea, the use of light could improve upon the number of cells effectively stimulated by the implant. Spiral ganglion cells expressing an activating opsin could be illuminated by a simple LED implanted locally and restore auditory function (DiGuiseppi and Zuo, 2019). The idea of repairing muscle paralysis with light is also appealing and promising results are already emerging. Functional optical stimulation has already been demonstrated in rodents and very recently the feasibility of light stimulation of peripheral motor nerves has been shown in NHP (Williams et al., 2019).

Applications in the treatment of Parkinson’s disease are also emerging through technologies based on neuromodulation such as opto-deep brain stimulation (Opto-DBS). Current DBS protocols are based on electrical stimulation delivered to a target brain area through a surgically implanted electrode. Despite being an approved therapy for Parkinson’s, the exact mechanism for DBS is not fully understood and protocols rely on clinical outcomes for optimization of the electrical strength and polarity of the neurostimulator. Another important issue with DBS is related to the absence of neuronal targeting during stimulation. Optical stimulation offers an attractive solution to this problem as it is possible to target the genetically encoded light-sensitive tools to particular cell types or a specific cellular compartment. Opto-DBS treatments would require the insertion of an optical probe delivering light to a large number of cells of which only a desirable fraction would respond (Lüscher et al., 2015; Gittis and Yttri, 2018).

Chronic pain continues to be one of the most common causes of disability that impairs quality of life. It remains difficult to treat; complete pain control with available drug treatment is rarely achieved and disabling side effects are common, including addiction, dependence, or even paradoxical hyperalgesia (Wang et al., 2012; Ferrini et al., 2013; Burma et al., 2017). In the context of the opioid crisis, non-pharmacological approaches for pain relief hold much therapeutic potential (Mickle and Gereau, 2018). While conventional electrical stimulation at the spinal level or in the skin show efficacy, the full potential of these approaches is not achieved because the stimulation approach is nonspecific and targets multiple cell types (e.g., different classes of sensory fibers during transcutaneous electrical nerve stimulation; different classes of afferents, local spinal interneurons, or ascending/descending pathways for spinal cord stimulation). Cell-specific optogenetic-based treatments for pain relief have been explored successfully in preclinical paradigms (Wang et al., 2016). Although far from being used in humans, strategies using an epidural optic fiber to deliver light to the spinal cord and sensory afferents expressing opsins are successful in mice (Bonin et al., 2016). Also, the use of miniature bio-optoelectronic implants to generate a closed loop of optoelectronic stimulation presents highly promising results in rodent models of bladder dysfunctions (Mickle et al., 2019). Translatability potential of the approach was also demonstrated by using viral transduction in dorsal root ganglion neurons *in vivo* (Spencer et al., 2018) but before these strategies can be safely used clinically, issues of transgene targeting remain to be completely solved.

## Choice of Gene Delivery Vehicle

As with other types of gene therapies, therapeutic applications of optogenetics will necessitate the expression of a genetically encoded protein in a specific cell type, organ or anatomical location and thus requires a delivery vehicle for the transgene. Such a targeted introduction of foreign genes is now done routinely in model organisms. However, translating such approaches to humans is, of course, associated with much higher ethical and safety standards and much lower tolerance of risk. Many approaches that are routinely used for transgenic animals, such as *in vivo* electroporation, are probably not translatable to humans. Other techniques, like the use of nanoparticles or carbon dots as gene carriers, show potential for therapeutic applications but research remains a relatively early stage (Zhou et al., 2016; Trigueros et al., 2019). Currently, viral vector-based transduction is the most advanced, powerful, and commonly used method to constitutively deliver foreign genes to specific tissues in mammals (Naso et al., 2017).

Viral vectors have a long track record in therapeutic gene delivery and research efforts are starting to bear even more fruit, as an increasing number of viral vector-based therapies are reaching the later stages of clinical studies (Keeler and Flotte, 2019). The approval of these strategies for the treatment of Lipoprotein Lipase Deficiency and hemophilia are landmark achievements of modern medicine (Gaudet et al., 2013; Chapin and Monahan, 2018). Amongst the many types of viral vectors, adeno-associated virus (AAV) is already being used in neural tissue to treat vision disorders (Bennett et al., 2016; FDA Briefing Document on Voretigene Neparvovec from Spark Therapeutics)^1^ and, based on current trends, is the frontrunner to be the method of choice for optogenetic applications in humans. However, even for AAVs, there remain major barriers to their widespread use in humans, including immune responses, specificity delivery/transport to the target cells, clearance of the vector through the liver, and the limited size of the gene cassette size which can be packaged in currently approved vectors.

For many clinical applications, lifelong expression of the optogenetic tool might be required and thus, maintenance of the transgene over time is an important consideration. AAV vectors do not consistently integrate their DNA into the host’s genome but persist episomally and have been shown to lead to prolonged gene expression with very low toxicity in various cell types including neurons (Gil-Farina and Schmidt, 2016; Hordeaux et al., 2019; Bravo-Hernandez et al., 2020). Although additional data is required, studies in rhesus macaques suggest that virally delivered opsins can remain functional for several months post-injection (Williams et al., 2019).

Other barriers to this fast-advancing field are the constraint of high regulatory scrutiny on production as well as the prohibitive costs associated with the use of patented gene delivery vectors. RegenxBio currently holds exclusive rights to most known AAV serotypes such as AAV7, 8, 9 and Rh10 and over 100 more through their NAV platform but other components such as the therapeutic transgene itself, and its mechanism of action (e.g., RNA interference, CRISPR) are often linked to licensing rights. Identifying exactly what intellectual property is owned by what inventor or institution and properly attributing rights and credit for all facets of potential gene therapy could be a complicated task. Obtaining the required rights can also quickly become costly and highly time-consuming (Kaemmerer, 2018).

One important constraint to the use of AAVs as gene delivery vehicles is the relatively small DNA packaging size. An AAV vector is limited to a single-stranded DNA cargo of approximately 5,000 bases (5 kb), which includes the necessary elements such as inverted terminal repeat (ITRs), polyadenylation sequence, and promoter. Generally speaking, most current optogenetic transgenes, fused to the gene encoding a fluorescent protein, span about 1.6–2.0 kb and thus fit within the rAAV constraints. As human applications may benefit from the combined use of multiple actuators (i.e., for two-color activation and silencing; Han and Boyden, 2007), or the combination of an optogenetic actuator and activity biosensors (Hochbaum et al., 2014), the size restriction of AAVs could limit their use as a delivery vehicle. Existing serotypes have been “over-packaged” with mixed success and varying reproducibility and the consensus appears to be that AAV can be overpackaged by ~10%, but with a concomitant reduction in both viral titers and *in vivo* transduction (Chamberlain et al., 2016). Trans-splicing is the favored approach currently used to increase the size of transgenes delivered through AAVs (Tornabene and Trapani, 2020). This approach relies on the splitting of the gene of interest and its separate packaging in two different vectors followed by their co-infection in the same cell. Since AAVs genomes will form concatemers, the two portions of the transgenes delivered separately usually end up being expressed as one gene (Colella et al., 2018). The challenge related to the implementation of such a strategy is the successful co-infection of the same target cell at levels high enough to obtain significant expression of the gene of interest. Molecular methods such as the use of protein trans-splicing mechanisms appear to increase the efficiency of the approach (Tornabene et al., 2019) but the issue remains particularly relevant when working with optogenetics tools which need to be expressed at relatively high levels to affect cellular processes.

Though not a technical constraint, another barrier to the use of currently available AAVs is restrictive multi-party intellectual property agreements resulting from the long chains of technical improvements made by different laboratories. Each contributor may impose intellectual property conditions that, collectively, preempt future developments. The challenge of costs thus relates to the development of new business models or funding mechanisms allowing for the development of these therapies as well as their usage within our health care systems.

## Gene Delivery and Targeting to The Tissue of Interest

Although other approaches have been reported (Dalkara et al., 2013), AAV-mediated delivery of optogenetic tools to the central and peripheral nervous systems of animal models has been mostly done with local injection. Relative to systemic administration, local injection avoids some of the issues described above. Indeed, immune responses are most problematic when the AAV is delivered into the bloodstream where it comes into direct contact with circulating antibodies. Neurosensory organs are particularly well suited for local administration of AAVs and monitoring of therapeutic effects. As such, ophthalmic disorders are among the most practical first targets for therapeutic optogenetics in humans.

In the central nervous system, the delivery of AAV through stereotaxic injection involves risks of viral or bacterial infection, hemorrhage, and edema. Also, as opposed to rodents for which detailed atlases of the brain exist and coordinates are well defined, surgical delivery in the human brain requires imaging and expert analysis immediately before the intervention which adds to the duration and cost of the process. The restricted spread of the injected vector can also be limiting, especially for the treatment of diseases that affect large areas of the CNS. Engineered AAV capsids such as the AAVDJ have shown increased spreading capacity with promising perspectives (Jollé et al., 2019). An alternative to stereotaxic injection is to deliver AAVs to the cerebrospinal fluid (CSF), which allows for more widespread gene transfer throughout the brain and spinal cord with a lower degree of precision required for injection (Hardcastle et al., 2018). However, the presence of tight junctions between ependymal cells can prevent AAV entry into brain parenchyma and will restrict such applications. In rodents, this technique has been used with the most success in newborns and young animals, which is also not applicable to humans (Hudry and Vandenberghe, 2019).

Due to the inherent challenges associated with stereotaxic injection and delivery *via* CSF, much effort has been directed towards the development of AAVs suitable for peripheral delivery in adult mammals. To obtain efficient targeting to the CNS from intravenous injections in adult mice, mutations have been introduced into the capsid of AAV9 to generate new serotypes such as PHP.B and PHP.S (Deverman et al., 2016; Chan et al., 2017; Challis et al., 2019). The efficiency of these engineered vectors for CNS targeting in mice has improved dramatically, but there is almost certainly further room for improvement. Initial efforts to use these serotypes in NHPs have demonstrated that these vectors do not exhibit the same targeting properties as observed in rodents and that toxicity is an issue (Hordeaux et al., 2018; Liguore et al., 2019). Indeed, with the high dose of vector required to achieve relevant transduction, adverse effects become significant. These results emphasize the importance of developing highly efficient viruses for human applications, such that the therapeutic window is associated with low viral titers. Hepatotoxicity represents a particularly significant risk as most AAVs are hepatotropic and considerable proportions of the vectors distribute to the liver. The most promising future direction to obtain the ideal delivery vehicle for optogenetics tools in the CNS is to undertake systematic testing in NHP to identify new vectors with the ability to cross the blood-brain barrier (BBB) while being excluded from various organs. Capsid engineering, *in vivo* selections, and directed evolution are all promising strategies for developing further improved AAV vehicles. An alternative could be the use of focussed ultrasound to disrupt the BBB and allow entry of AAVs into the brain (Chen et al., 2019). Fortunately, the natural biological diversity of AAV serotypes, which is diverse and displays remarkable differences in gene transfer and vector tropism between serotypes, remains a rich resource that should continue to be mined to discover improved tools for gene delivery.

Although a broad AAV biodistribution throughout the CNS could be desirable for some applications, targeting specific anatomical areas or cell types is often important for the specificity of treatment. Efficient long-distance anterograde and retrograde axonal transport of certain AAV serotypes has been demonstrated in various animal models (Tervo et al., 2016; Zingg et al., 2017). Assuming that there is a connected area that is easily reachable for local injection and associated with minimal risks of infection, this type of transport could be used as a means to promote the introduction of viral particles, and their payloads, across anatomically connected areas of the brain. However, considering that our knowledge of the human neuronal connectome remains relatively sparse, it is unlikely that this strategy will be applicable on a large scale shortly. Of course, one of the main advantages of optogenetics is that activation requires both gene delivery and illumination. Accordingly, even in cases where tissue-specific gene delivery is imperfect or not possible, spatially confined illumination will provide a means of activating specific tissue areas.

Beyond general tissue specificity, AAV serotypes can exhibit unique cellular tropisms, though there is still much progress that needs to be made to achieve high levels of cell-type specificity. Indeed, despite numerous studies reported on the life cycle of AAVs, the molecular basis of the varied tropisms of AAV vectors is still being elucidated (Srivastava, 2016). Although most natural AAVs have been shown to use cell surface glycans as primary receptors, structure differences in both the AAV capsids and receptor glycans have been linked to variations in transduction efficiencies and tropism (Asokan et al., 2012; Murlidharan et al., 2014). A recently identified cellular receptor (AAVR) has been shown to bind most AAVs (Summerford et al., 2016) but the role, if any, that this receptor plays in larger animals and humans remains to be shown. Interestingly, the identification of a new class of receptors that binds the engineered PHP-B capsids (Huang et al., 2019) independently of previously known receptors, may improve our understanding of variations in tropisms between species and provide new ways to exploit the use of cellular receptors in AAV targeting.

Another approach to achieving cell-type specificity in rodents and NHPs is to drive optogenetic tool expression using cell-specific eukaryotic promoters (Galvan et al., 2017). Inherent challenges for promoter development are the contradictory requirements for small size (constrained by the packaging limitations of the AAV capsid) and high expression level (required to impact cell function). Although initiatives such as the “Pleiades promoter project” have contributed to the elaboration of databases of mini-promoters with potential for human brain gene therapy (de Leeuw et al., 2014), only a few are well characterized and drive expression levels strong enough for the needs of optogenetics applications. Our ever-increasing understanding of gene regulation has led to the emergence of optimized “tailor-made” expression cassettes with improved efficacy which are exploiting strategies such as miRNA or nucleotide structure-based control (Papadakis et al., 2004; de Leeuw et al., 2014; Domenger and Grimm, 2019; Zhong et al., 2020). A promising strategy is the addition of strong distal enhancer elements upstream of the core-specific promoter which can increase the level of transgene expression while maintaining a small size promoter and the tissue specificity (Blankvoort et al., 2018, 2020). Challenges remain, however, in the translation of these developments to humans. Although it is known that the same promoters can drive gene expression in different animal species, the specificity of a given promoter can vary between species.

While there remains a tremendous potential for engineered promoters for therapeutic applications, detailed characterization of *in vivo* expression patterns in humans seems impractical or impossible. For this reason, the use of human-induced pluripotent stem cells or cerebral organoids (Shiri et al., 2019) to validate transduction efficiency driven by cell-specific promoters could be considered. Though, they are far from perfect models of *in vivo* biology, these quickly evolving technologies are extremely promising and should be included in promoter and vector validation pipelines.

## Immunogenicity and Genotoxicity

Human optogenetic-based therapies, like many other cell-, gene-, and protein-based therapies, will be complicated by the possibility of immune responses. The introduction and expression of foreign molecules, such as a light-activated opsin, comes with the risk of eliciting a response from our immune cells. Furthermore, the AAV delivery vehicle itself could also be targeted by our immune system through pre-existing immunity (PEI; Bartel et al., 2011). PEI occurs due to prior exposure to an AAV, which introduces a lasting memory immune response. Since AAV is endemic in humans and most AAV serotypes currently in use came from primates, most patients will have likely been pre-exposed and may carry circulating anti-AAV neutralizing antibodies secreted by memory B cells. As these antibodies can, even at low levels, prevent the AAV particle from reaching its intended tissue or cell target, PEI needs to be considered when developing AAV based therapies (Meadows et al., 2019; Nidetz et al., 2019).

To develop AAVs that are minimally or negligibly inactivated due to PEI, it might be necessary to develop AAV vectors from isolates and serotypes not found in humans or NHP and with sequence differences significant enough that the circulating antibodies would not recognize and neutralize the viral particles. Alternatively, as regions of the AAV capsid important for antibody binding have been identified, inserting mutations in these residues could abolish epitope recognition (Kotterman and Schaffer, 2014), or the AAV could be pegylated to shield the virus capsid from the immune system (Yao et al., 2017). Strategies involving the manipulation of the host may also be considered. For example, using plasmapheresis, which could remove most antibodies against AAV, or suppressing innate immunity could potentially mitigate the effect of a possible antibody response (Tse et al., 2015).

In addition to potential immune responses against the viral delivery vehicle, immune responses against the optogenetic protein itself are a major concern. Notably, microbial opsins are membrane-spanning proteins so they necessarily present non-human epitopes to the extracellular environment. This hypothesis has so far been mostly examined in the context of vision restoration therapy (Sugano et al., 2011, 2016). It was shown that in the retina, which is a known immune-privileged site, the specific reaction to ChR2 or mVChR1 is minimal and does not affect its expression. However, when Maimon et al. (2018) recently tested the immunogenicity of ChR2 following intramuscular injection in rats (AAV delivery), significant levels of anti-ChR2 antibodies were detected in the serum. More importantly, they observed loss of expression and cell death induced by the immune reaction, suggesting that researchers should be cautious when using optogenetic tools of microbial origins. Protein engineering to remove the most immunogenic epitopes or “humanize” opsin proteins may be one approach to addressing this challenge. Yet another approach would be to re-purpose human opsins as optogenetic tools. Proof-of-concept for this approach comes from the work of Berry et al. (2019), who restored vision in a mouse by AAV-mediated delivery of the gene for mammalian medium wavelength cone opsin (MW-opsin) in the retina.

In addition to immunogenicity, close attention must be paid to the possibility of AAV gene delivery-induced mutagenicity or genotoxicity that could serve as a driver for tumor formation. Fortunately, relative to other potential viral delivery vehicles, AAV-delivered transgenes rarely insert into the host’s genomic DNA (Colella et al., 2018). Rather, the AAV-delivered DNA cargo tends to exist as a nuclear-localized circular double-stranded DNA known as an episome. Accordingly, AAVs have relatively low mutagenicity or genotoxicity. Even though the insertion rate is low, the fact that AAVs used for therapeutic purposes would likely deliver very strong promoters to drive high-level expression, even rare genomic integration events could drive increased expression levels of adjacent oncogenes.

## Improved Optogenetic Tools of Potential Utility for Therapeutic Applications

In parallel with efforts to develop improved gene delivery methods, ongoing efforts are providing improved optogenetic tools with higher light sensitivity and sensitivity to longer wavelength (lower energy) light. To realize these improvements, researchers have used multiple approaches including mutagenesis, chimera-genesis, and discovery of improved genes in nature. For example, early efforts to further improve the light-gated cation channel channelrhodopsin-2 (ChR2) produced several variants with higher light sensitivity, larger photocurrent, and/or faster kinetics (Lin et al., 2009; Gunaydin et al., 2010; Berndt et al., 2011). As another example, a bioinformatic search against the sequenced genome of cryptophyte *Guillardia theta* led to the discovery of light-activated opsins that are highly light-sensitive and efficiently hyperpolarize the membrane and silence the neuron through anion conduction (Govorunova et al., 2015). More recently, machine learning has been applied to guide opsin engineering to simultaneously optimize multiple properties including localization, kinetics, photocurrents, and light sensitivity (Bedbrook et al., 2019).

**Figure 1:**
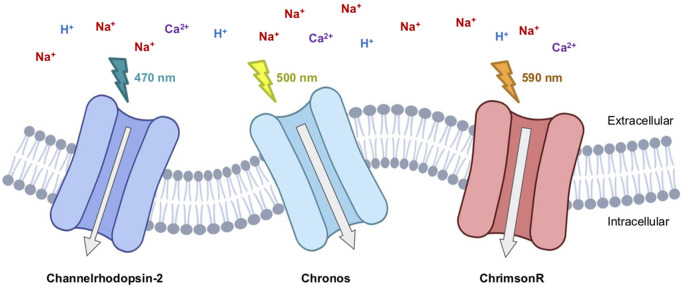
Therapeutic-relevant opsins currently involved in clinical trials. Channelrhodopsin2 (Clinical Trial #NCT02556736), Chronos (Clinical Trial #NCT04278131), and ChrimsonR (Clinical Trial #NCT03326336) are excitatory light-gated cation channelrhodopsins.

In addition to efforts to increase the light sensitivity, substantial efforts have been invested in the discovery and engineering of optogenetic tools with higher sensitivity to red-shifted wavelengths of light. The use of more red-shifted activation light is associated with reduced potential for tissue photodamage and greater tissue penetration of the light. The red-shifted channelrhodopsin variant VChR1 (~550 nm activation peak) was first identified from *Volvox carteri* using a genomic screening strategy. However, weak expression and small photocurrents initially limited its practical utility (Zhang et al., 2008). A chimeric opsin variant, C1V1, composed of sequences from both ChR1 and VChR1, retained the red-shifted spectrum but also exhibited improved membrane trafficking and enhanced photocurrents (Yizhar et al., 2011). A further red-shift was realized with the ReaChR variant (~600 nm activation peak) which was also engineered using chimera-genesis and rationally designed mutations (Lin et al., 2013). In the ongoing search for further improved red-shifted opsins with large photocurrents and high light-sensitivity, genome screens have helped to identify new variants such as Chrimson from *Stigeoclonium helveticum* (Klapoetke et al., 2014), and ChRmine from *Tiarina fusus* (Marshel et al., 2019). GenSight Bioscience is currently performing a clinical trial (NCT03326336) in which a Chrimson variant is being explored for vision restoration (Supplementary Table S1; Figure 1).

The issue of the alteration of ion balance which can be induced by the activity of certain opsins should also be considered when designing tools for therapeutic applications (Wang et al., 2016). It is known that inhibitory opsins, which have mostly been based on ion pumps (Cl^−^ for NpHR; H^+^ for Arch and ArchT) may result in undesirable side effects such as the collapse in Cl^−^ gradients and pH changes. Rebound excitation, due to the accumulation of Cl^−^ ions following the end of NpHR activation, for example, has been found (Gradinaru et al., 2007; Raimondo et al., 2012). This caveat may be further compounded in disorders associated with Cl^−^ imbalance such as chronic pain (Coull et al., 2003), drug dependence (Ferrini et al., 2013; Ostroumov et al., 2016), and certain symptoms of autism (Anacker et al., 2019). In this context, the recent advent of Cl^−^ permeable opsins may be more promising (Govorunova et al., 2015; Wietek et al., 2015).

## Light Delivery

Light delivery is at the heart of the technical challenges of human optogenetics: the sampling volume within which the irradiance is sufficient for stimulation is essentially the same in rodents and humans because the tissue properties are similar. However, the number of neurons that need to be excited in humans is larger than in rodents, and therefore the volume of tissue that must be illuminated is also larger. Therefore, the strategies that have worked in rodents (e.g., point excitation from multimode fibers) are likely going to be insufficient to trigger a response in humans. There are two options to maximize the number of illuminated neurons: the use of volume diffusers or longer wavelength illumination. Here, we can learn from other fields where light delivery to large volumes is also a challenge. For example, photodynamic therapy must also maximize the excitation volume for tumor treatment and many solutions have been proposed such as fiber diffusers (Mizeret and van den Bergh, 1996; Selm et al., 2007) or multi-fiber geometries. In Diffuse Optical Tomography, reflectance measurements are routinely performed up to a few centimeters through the skull thanks to the use of infrared light (800 nm; White et al., 2009). The required power for photodynamic therapy and tomography experiments is lower than for optogenetics stimulation, but they provide additional arguments for red-shifted channels. In as much as the volume of excitation is maximized, we still required a minimum irradiance to generate an action potential: when local expression levels are sufficient, the threshold irradiance is nominally 10 mW/mm^2^ for ChR2 in rodents (Boyden et al., 2005), which can be achieved with LEDs or lasers alike fairly easily, even with wirelessly charged μ-LED (Shin et al., 2017). However, it is not trivial to estimate the necessary power that will be sufficient for humans: even if several milliwatts of power are incident on the tissue, it will be quickly redistributed due to scattering and will be removed from the tissue with absorption.

Although, it is outside the scope of this review article to explain the details of light scattering calculations in tissue, we can still provide important guidelines and even tools for the interested researchers. The light distribution within tissue depends on the scattering and the absorption coefficients, two properties that depend on the light wavelength and that are difficult to characterize accurately. In addition to the uncertainty in their exact values, the random scattering and absorption processes lead to a light distribution that is a rapidly varying function of space with intricate reflections from surrounding tissue. The reduced scattering coefficient (i.e., the scattering coefficient multiplied by (1-g) where g is the anisotropy factor) ranges from 10 cm^−1^ to 100 cm^−1^ in nervous tissue and varies only mildly with a wavelength in the visible (Cheong et al., 1990). On the other hand, the absorption coefficient is essentially zero above a wavelength of 600 nm but is over 1,000 cm^−1^ due to hemoglobin absorption around 488 nm. The great variability and the large values of these coefficients make the final distribution of light very difficult to estimate accurately. The tools used to perform such estimates are Monte Carlo simulations, with the most common and validated software being MCML (Wang et al., 1995). Today, it is even possible to use web-based calculations to obtain reasonable estimates sufficient to choose a laser adequately (Doronin and Meglinski, 2011). It is expected that the typical illumination volume from a point source in the brain will increase from 1 mm^3^ to 1 cm^3^ (a thousandfold increase) when going from 488 nm to 600 nm excitation wavelength (DePaoli et al., 2020), indicating that other solutions such as volume diffusers may be necessary to trigger a response if red-shifted channels are not available.

## Conclusions

**Figure 2:**
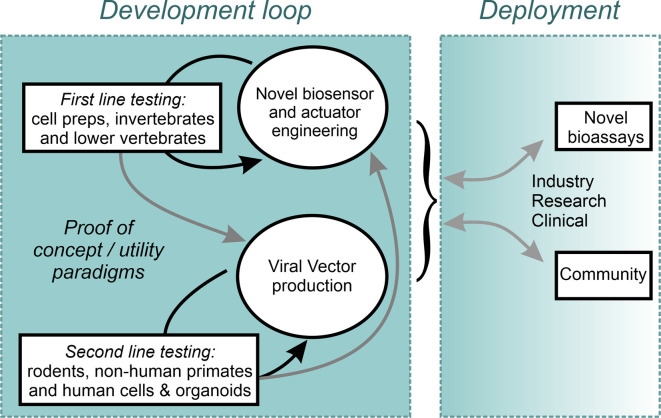
An open science Design-Build-Test model implemented within the Canadian Neurophotonics Platform, giving rise to the Canadian Optogenetics and Vectorology Foundry project (neurophotonics.ca).

Ultimately, to enable the most sophisticated levels of neuronal control, the combined use of optogenetic actuators and genetically encoded biosensors of cellular activity indicators could enable closed-loop all-optical activity neuromodulation. Such systems would introduce additional challenges related to spectral orthogonality between actuator and indicator, the need for decision making computational algorithms, and the need to deliver large gene cassettes. As highlighted above, there is not a single strategy which appears to fulfill all the requirements for the ideal delivery method of optogenetics tools in humans. As such, likely, combining multiple approaches (light, serotype, delivery route, promoter, etc.) will be needed to obtain the proper level of targeting and cellular specificity. Moving the field forward will require researchers to address an extremely large number of challenges that will only be overcome by testing in NHP as well as human *in vitro* models. This monumental task will far exceed the capabilities of any one team and will succeed only if a proper structure of data sharing is put in place and the whole of the vested research community is compelled to contribute. A proposal to accelerate this effort is the rapid, unencumbered, dissemination of open-source constructs, combined with open communication of both positive findings and setbacks. An example of such an enterprise is the effort developed within the Canadian Neurophotonics Platform project (Figure 2; neurophotonics.ca). Through the combined efforts and transdisciplinary expertise of the research community, therapeutic applications of optogenetics no longer need to be “just over the horizon,” and can be made into a therapeutic reality in the here and now.

## Author Contributions

YS contributed the entire text related to improved genetic tools for therapeutic applications. RC contributed to all sections of the text and M-EP initiated the project and wrote sections pertaining to viral vectors and near-future prospects for therapeutic applications. DC wrote the section on light.

## Conflict of Interest

The authors declare that the research was conducted in the absence of any commercial or financial relationships that could be construed as a potential conflict of interest.

## Footnotes

^1^www.fda.gov/media/108375/download
