# Supplementary material for: Challenges for Therapeutic Applications of Opsin-Based Optogenetic Tools in Humans
Source: Front Neural Circuits. 2020 Jul 15;14:41. doi: 10.3389/fncir.2020.00041 (PMC7373823; doi:10.3389/fncir.2020.00041)
Supplement: Supplementary file 1 [file Data_Sheet_1.PDF]

Table 1. Therapeutic relevant opsins (opsins currently involved in clinical trials)

Opsin	Species	Peak λ	Permeability	Clinical Trial #	Size
Channelrhodopsin2	*Chlamydomonas reinhardtii*	470 nm	Cation	NCT02556736	945 bp
Chronos	*Stigeoclonium helveticum*	500 nm	Cation	NCT04278131	978 bp
ChrimsonR	*Chlamydomonas noctigama*	590 nm	Cation	NCT03326336	1056 bp
